# Melatonin reduces the endoplasmic reticulum stress and polyubiquitinated protein accumulation induced by repeated anesthesia exposure in *Caenorhabditis elegans*

**DOI:** 10.1038/s41598-022-09853-y

**Published:** 2022-04-06

**Authors:** Hyun-Jung Shin, Bon-Wook Koo, Jiwon Yoon, Heeyeon Kim, Sang-Hwan Do, Hyo-Seok Na

**Affiliations:** Department of Anesthesiology and Pain Medicine, Seoul National University Bundang Hospital, Seongnam, Gyeonggi 13620 South Korea

**Keywords:** Neuroscience, Medical research, Molecular medicine

## Abstract

Endoplasmic reticulum (ER) stress has been linked to anesthesia-induced neurotoxicity, but melatonin seems to play a protective role against ER stress. Synchronized *Caenorhabditis elegans* were exposed to isoflurane during the developmental period; melatonin treatment was used to evaluate its role in preventing the defective unfolded protein response (UPR) and ER-associated protein degradation (ERAD). The induced expression of *hsp-4*::GFP by isoflurane was attenuated in the isoflurane-melatonin group. Isoflurane upregulated the expression of *ire-1*, whereas melatonin did not induce *ire-1* expression in *C. elegans* even after isoflurane exposure. With luzindole treatment, the effect of melatonin on the level of *ire-1* was significantly attenuated. The reduced expression of *sel-1*, *sel-11, cdc-48.1,* and *cdc-48.2* due to isoflurane was restored by melatonin, although not up to the level of the control group. The amount of polyubiquitinated proteins was increased in the isoflurane group; however, melatonin suppressed its accumulation, which was significantly inhibited by a proteasome inhibitor, MG132. The chemotaxis index of the isoflurane-melatonin group was improved compared with the isoflurane group. Melatonin may be a potential preventive molecule against defective UPR and ERAD caused by repeated anesthesia exposure. The *ire-1* branch of the UPR and ERAD pathways can be the target of melatonin to reduce anesthesia-induced ER stress.

## Introduction

Anesthetic agents have undeniably been used very safely during the last half of the century. Nevertheless, they are currently being associated with previously unrecognized neurotoxic effects that manifest at an advanced age or after multiple exposures. Over the past decade, volatile anesthetic agents have been reported to trigger apoptotic neuronal degeneration and learning deficits in neonatal animals and primates^1–4^. Therefore, a better understanding of anesthesia-induced neurodegeneration and determination of its importance in human medicine have become imperative. Additionally, understanding the anesthetic mechanism is an obligation in order to circumvent its toxic effects in vulnerable patients.

The crucial role of endoplasmic reticulum (ER) stress has previously been reported with regard to anesthesia-induced neurodegeneration^5–8^. Isoflurane has been shown to cause ryanodine receptor-associated ER stress, resulting in caspase-3 activation, and eventually neurotoxicity^5^. Sevoflurane-mediated ER stress was found to be associated with hippocampal injury^6^. ER stress is characterized by the accumulation of misfolded proteins. It activates the unfolded protein response (UPR), which is comprised of three branches: the ribonuclease inositol-requiring proteins-1 (IRE-1), the protein kinase RNA-like endoplasmic reticulum kinase (PERK), and activating transcription factor-6 (ATF-6)^9^. ER-associated protein degradation (ERAD) is a major pathway associated with the translocation of misfolded proteins from ER lumen into the cytosol for subsequent ubiquitination, which allows them to be degraded by proteasomes^9^. The UPR and ERAD are well conserved between mammals and *C. elegans*^10^. Recently, we found ER stress and UPR to be increased and ERAD interrupted by repeated isoflurane exposure, thereby causing the accumulation of ubiquitinated proteins in *C. elegans*^11^.

Melatonin (N-acetyl-5-methoxytryptamine) is synthesized primarily by the pineal gland in mammals and regulates the circadian rhythm. Previous studies have reported melatonin to have anti-oxidative properties^12,13^ and free-radical scavenging effects^14^. In addition, its protective action against ER stress through the activation of the ERAD pathway had been proposed earlier^15^. In this study, we aimed to investigate whether melatonin can modulate the UPR and ERAD pathways induced by repeated anesthesia, and prevent the accumulation of ubiquitinated proteins in *C. elegans*.

## Results

### Effect of melatonin on ER stress

**Figure 1:**
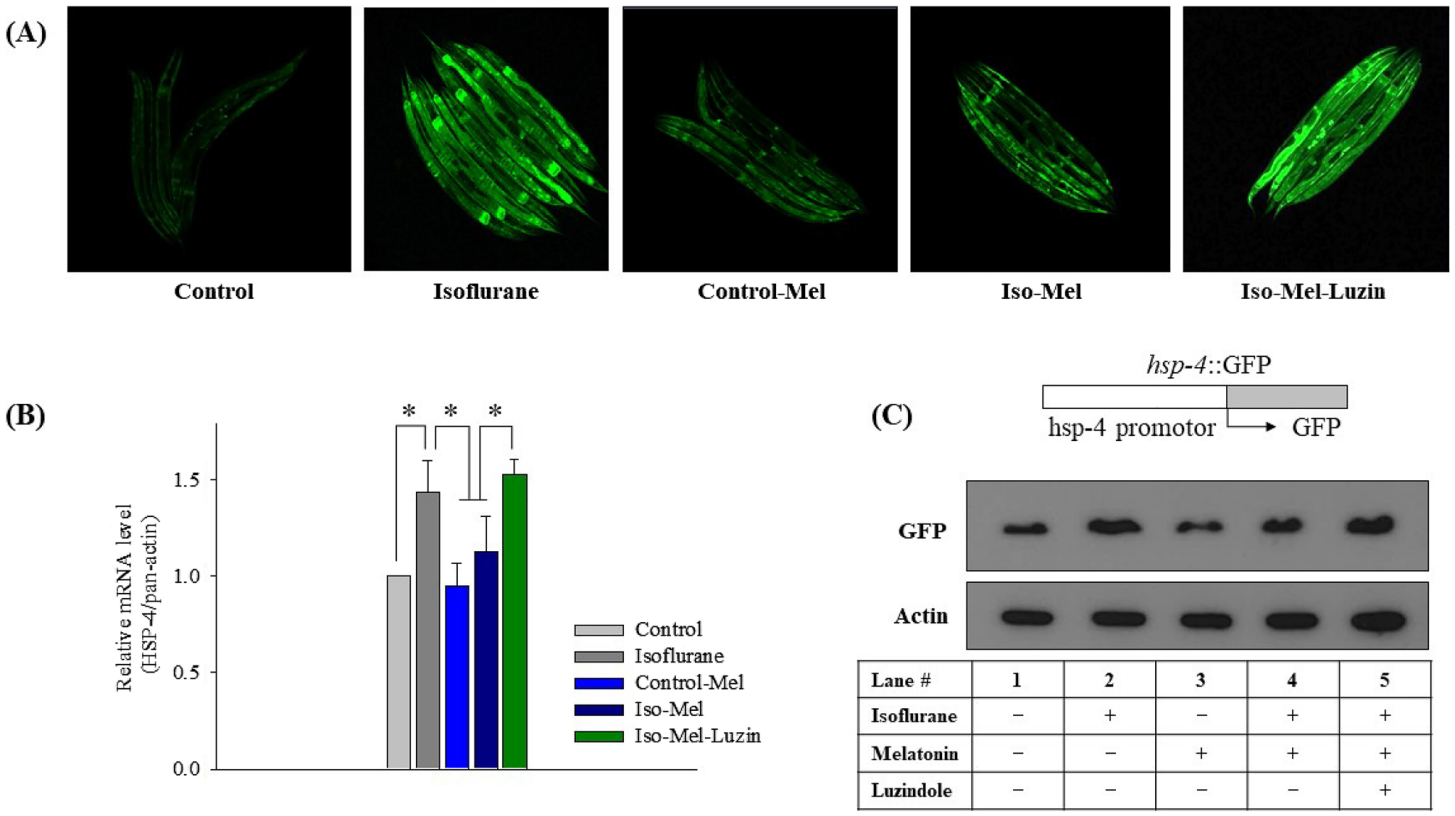
*Phsp-4::GFP* expression in young adults after repeated isoflurane exposure with or without concurrent melatonin or luzindole treatment. (**A**) *Phsp-4::GFP* expression. *Phsp-4::GFP* expression was increased significantly after isoflurane exposure, while it was decreased with concurrent melatonin treatment. The inhibitory effect of melatonin on *Phsp-4::GFP* expression was significantly suppressed by luzindole. The experiments measuring GFP were performed 5 times and 5–10 worms per condition were monitored in each experiment. (**B**) Expression of the *hsp-4* gene. All batches included three plates in each group, and the same assay was performed three times. (**C**) Schematic diagram of the *hsp-4::*GFP reporter construction and western blot for GFP expression. Western blot analysis was performed by using equal amounts of protein lysate prepared from approximately 500 worms. Error bar, standard deviation; *P < 0.05 *vs*. the control group; ^†^P < 0.05 *vs*. the control-melatonin group; ^‡^P < 0.05 *vs*. the isoflurane-melatonin group.

In *C. elegans*, *hsp-4* was monitored to evaluate ER stress, it being homologous to binding immunoglobulin protein (BiP) in humans. The expression pattern of *hsp-4*::GFP is shown in Fig. 1A. The induced expression of *hsp-4*::GFP by isoflurane was attenuated in the isoflurane-melatonin group. A melatonin receptor antagonist, luzindole, was added during the isoflurane exposure period, and it abolished the effects of melatonin on *hsp-4*::GFP. The changes of *hsp-4* gene level and GFP protein in each group were validated by real time-PCR (Fig. 1B) and western blot of GFP (Fig. 1C), respectively.

### ER_UPR_ and ER-associated protein degradation by melatonin

To determine the effects of melatonin on the regulation of UPR, representative UPR genes, *ire-1*, *pek-1*, and *atf-6* of *C. elegans* (corresponding to human IRE1, PERK, and ATF6) were studied by real-time PCR.

**Figure 2:**
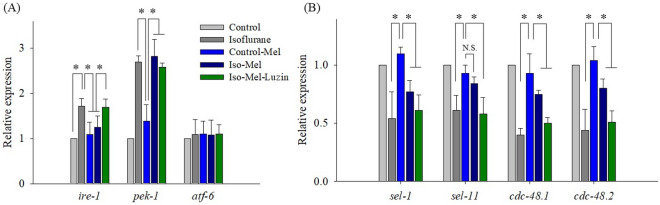
Expression of genes related to the unfolded protein response and endoplasmic reticulum-associated protein degradation pathways. (**A**) Increased *ire-1* expression followed by repeated isoflurane exposure was suppressed by melatonin treatment. Luzindole attenuated the effect of melatonin on the level of *ire-1*. (**B**) The expression of *sel-1*, *sel-11*, *cdc-48.1*, and *cdc-48.2* was significantly decreased after repeated isoflurane exposure. While the differences in *sel-1, cdc-48.1,* and *cdc-48.2* expression were still significant between the control-melatonin and the isoflurane-melatonin groups, *sel-11* expression was comparable between the two groups. All batches included three plates in each group, and the same assay was performed three times. Error bar, standard deviation; *P < 0.05; N.S., not significant.

Isoflurane upregulated the expression of *ire-1* (P < 0.001), whereas melatonin did not induce *ire-1* expression in *C. elegans* even after isoflurane exposure (P = 0.156 *vs.* the control group). When luzindole was added, it significantly attenuated the effect of melatonin on the level of *ire-1* (Fig. 2A). Although *pek-1* was enhanced by isoflurane (P < 0.001), melatonin or luzindole had no effect on its level (P < 0.001 *vs.* the control group and P = 0.508 *vs.* the isoflurane group). Moreover, the expression of *atf-6* was not affected by isoflurane, melatonin, or luzindole.

Figure 2B shows the relative expression of four genes related with the ERAD pathway. Isoflurane downregulated *sel-1*, *sel-11, cdc-48.1,* and *cdc-48.2* expression significantly, while melatonin alone had no effect on them. Reduced expression of *sel-1*, *sel-11, cdc-48.1,* and *cdc-48.2* caused by isoflurane was restored by melatonin, although not up to the level of the control group. The most protective effect of melatonin was observed in *sel-11*. While the differences of *sel-1, cdc-48.1,* and *cdc-48.2* expression were still significant between the control-melatonin and the isoflurane-melatonin group, *sel-11* expression did not decrease significantly by combined exposure of isoflurane and melatonin. However, luzindole treatment significantly eliminated the effect of melatonin on *sel-1*, *sel-11, cdc-48.1,* and *cdc-48.2* expression.

### Accumulation of polyubiquitinated proteins by melatonin

**Figure 3:**
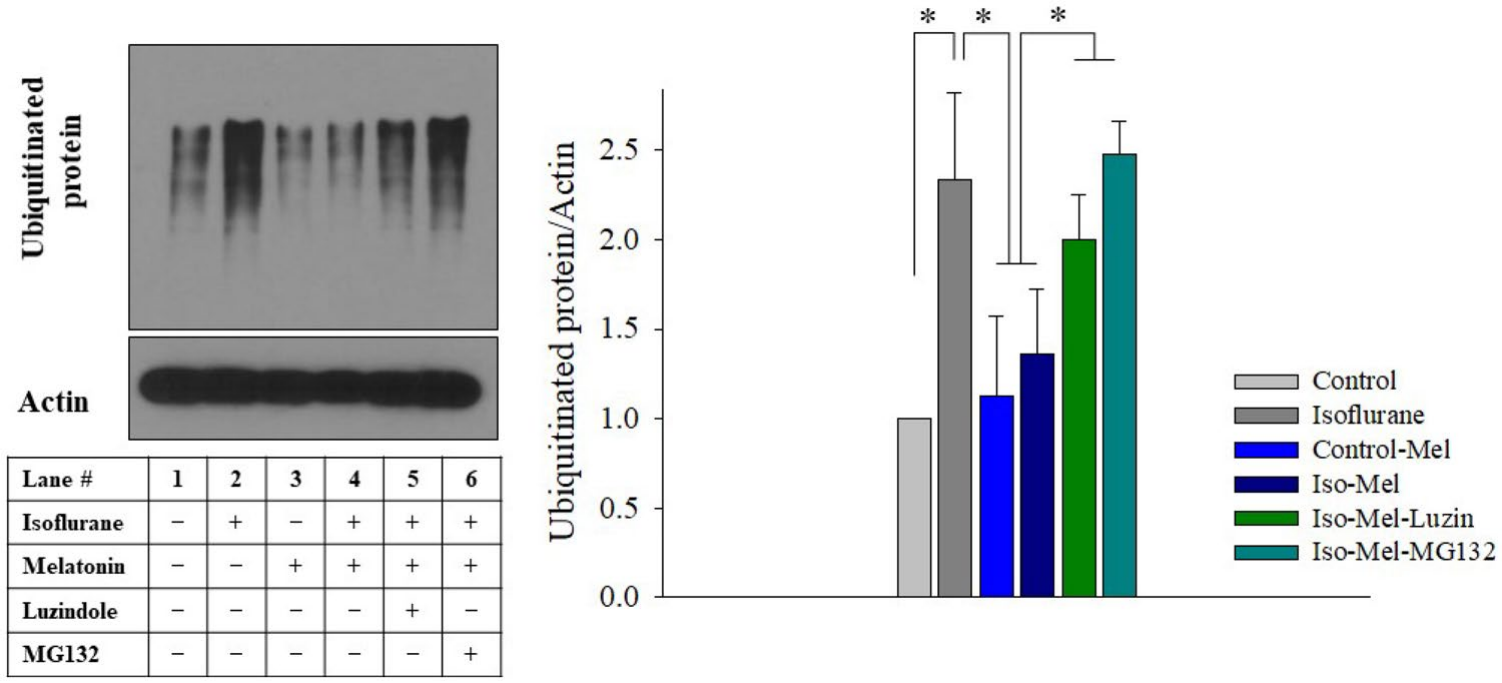
Western blot for ubiquitinated proteins. Higher levels of polyubiquitinated protein were observed in the isoflurane group, which was attenuated by melatonin treatment. The inhibitory effects of melatonin on the accumulation of ubiquitinated proteins were significantly suppressed by luzindole and a proteasome inhibitor, MG132. All batches included three plates in each group and the same assay was performed five times. Error bar, standard deviation. *P < 0.05.

When the ERAD pathway is disturbed by isoflurane, unfolded or misfolded proteins can accumulate in the ER or cytoplasm. Misfolded proteins are ubiquitinated in the cytoplasm before their removal by proteasome for degradation. The amount of polyubiquitinated proteins was increased in the isoflurane group (P < 0.001), but melatonin suppressed its accumulation (P = 0.040 *vs.* the control group) (Fig. 3). The inhibitory effects of melatonin on the accumulation of ubiquitinated proteins were significantly suppressed by luzindole and a proteasome inhibitor, MG132.

### Effect of melatonin on chemotaxis index by isoflurane

**Figure 4:**
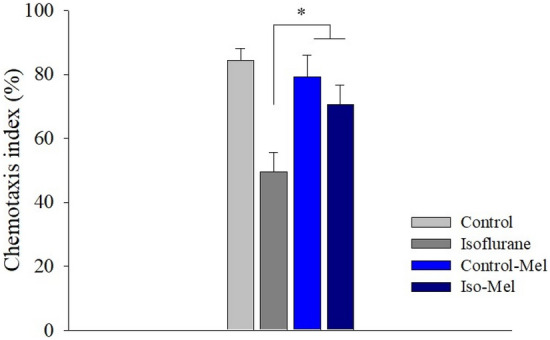
The chemotaxis index following the melatonin treatment. When *C. elegans* was not treated with melatonin, the chemotaxis index decreased significantly following isoflurane exposure. Melatonin treatment during the isoflurane exposure period could restore the chemotaxis index. *P < 0.05.

The chemotaxis index was decreased significantly due to repeated isoflurane exposure, as previously reported (P < 0.001)^11,16^. When *C. elegans* was grown in NGM plates containing melatonin, the chemotaxis index was not significantly affected (P = 0.624). The chemotaxis index of the isoflurane-melatonin group was better than that of the isoflurane group (P < 0.001); however, it was still significantly lower than that of the control group (P = 0.007) (Fig. 4).

## Discussion

Various studies have demonstrated that prolonged ER stress is related to neurodegenerative diseases, such as Parkinson’s disease or Alzheimer’s disease; neuronal apoptosis and neuroinflammation are caused by a persistent and excessive stress response^17,18^. Inhaled anesthetics have been reported to cause ER stress, which may be related to anesthesia-induced neurotoxicity (AIN)^6,7,19,20^. In our previous report, we found that repeated inhalation anesthetics could cause significant ER stress with UPR and ERAD pathway disruption followed by ubiquitinated protein accumulation in a *C. elegans* model^11^.

The role of melatonin in the prevention or treatment of PD or AD has been discussed earlier^21,22^. However, whether melatonin modulated ER stress and ERAD pathway in AIN was not clear. To the best our knowledge, this study is first to show that melatonin modulates the expression of *ire-1* and prevents the depletion of ERAD-related genes in isoflurane-exposed *C. elegans*. Specifically, the accumulation of polyubiquitinated proteins was reduced by melatonin.

Melatonin significantly attenuated the isoflurane-induced upregulation of *hsp-4*, which is the worm homologue of BiP. In *C. elegans*, *hsp-4* acts as an important sensor of ER stress and is a key upstream component of UPR^23^. When ER stress is triggered, *hsp-4* is dissociated from the luminal domain of *ire-1* or *pek-1* and binds to the unfolded proteins. Consequently, the *ire-1* and *pek-1* branches of UPR initiate their role in managing ER stress^24,25^. Of these two genes, melatonin modulated the expression of *ire-1* only. Accordingly, we proposed that the *ire-1* pathway, one of the UPR signaling pathways, might play an important role in melatonin-related neuroprotection in *C. elegans* under anesthesia-induced ER stress. However, it remains unclear whether melatonin might directly reduce the *hsp-4* or *ire-1* expression. IRE1 phosphorylation and subsequent XBP1 splicing in anesthesia-induced neurodegeneration, and melatonin’s protective role should be evaluated in further studies.

Misfolded proteins are usually retrotranslocated to the cytosol, where the ones that are ubiquitinated are degraded by the proteasome^26^. The ERAD complex formed by SEL1L and HRD1 is conserved in mammals, and is known to be related with neurodegenerative diseases such as Parkinson’s or Alzheimer’s disease^27–32^. In *C. elegans*, their orthologs, *sel-1* and *sel-11*, were affected by repeated isoflurane exposure^11^. In addition, p97 is known to expedite the degradation of misfolded proteins. The central component of the ubiquitin–proteasome system is p97, which guides protein substrates to the 26S proteasome for degradation^33^. *C. elegans* uniquely possesses two p97 homologues, namely *cdc-48.1* and *cdc-48.2,* both depleted by repeated isoflurane exposure^11^.

Melatonin treatment prevented the significant depletion of these four ERAD-related genes after repeated isoflurane exposure, even though they did not reach the level of the control group. Interestingly, the expression of *sel-11* only did not show a significant difference after isoflurane exposure in melatonin-treated worms. Together with *ire-1* in UPR, *sel-11* was the gene that benefited the most from the protection of the melatonin treatment in *C. elegans.* Previously, the close relationship between IRE1 and HRD1 was reported in humans, where ER stress induces HRD1 expression through the IRE1 pathway to maintain homeostasis^34,35^. Treatment with melatonin also reduced the levels of polyubiquitinated proteins by maintaining ERAD function. Furthermore, a proteasome inhibitor, MG132, significantly suppressed the melatonin effect on ubiquitinated protein accumulation under ER stress. Thus, under ER stress, melatonin might regulate ubiquitinated protein levels through the ERAD system.

Evidence has been found of the accumulation of polyubiquitinated proteins in neurodegenerative diseases^36–38^. The effects of melatonin have previously been examined in order to identify potential therapeutic or preventive agents in several neurodegenerative diseases. For example, the pathological signature of Alzheimer’s disease includes the deposits of Aβ plaques and neurofibrillary tangles, which promote neuronal degeneration; melatonin treatment was found to enhance the clearance of Aβ in Alzheimer’s disease transgenic mice^39–41^. Secondly, the accumulation of aggregated mutant α-synuclein leads to the formation of intracellular inclusions called Lewy bodies, which are the major hallmark of Parkinson’s disease^42^. Melatonin could attenuate the expression of α- synuclein in the dopaminergic pathway and protect neurons from α-synuclein-induced cytotoxicity^43,44^. The results led to the hypothesis that melatonin could specifically reduce the accumulation of abnormal protein aggregation via the activation of ERAD. Unfortunately, we cannot reveal the characteristics of accumulated polyubiquitinated proteins yet. Tao et al. reported that multiple exposure to inhalation anesthetics induces Tau phosphorylation and cognitive impairment in mice^45^. In addition, several commonly used anesthetics might increase Aβ accumulation in animal models^46^. Given the limited profiles about the accumulated pathological proteins due to anesthetic agents, targeted protein analysis is warranted in the future.

Melatonin shows an effect through the G protein-mediated MT1, MT2, or MT3 receptors, and it regulates neural activities through MT1 receptors in *C. elegans*^47^. In this study, luzindole significantly suppressed the effects of melatonin on *hsp-4*, *ire-1*, *sel-11*, and ubiquitinated protein accumulation. Thus, we speculate that melatonin may play a protective role in AIN via MT1 receptors. Additional research is required on the details of molecular and genetic mechanisms of melatonin in defective UPR and ERAD caused by repeated anesthesia exposure.

In a *C. elegans* model, AIN was proved by a chemotaxis assay. We have previously reported that repeated isoflurane exposure decreases the chemotaxis index^16,48^, correlated with ER stress and ERAD abnormalities^11^. Based on what has been discussed above, melatonin seemed to prevent AIN in *C. elegans*, which was proved by the recovered chemotaxis index even after repeated isoflurane exposure.

In conclusion, our results suggest that melatonin may be a potential preventive molecule against defective UPR and ERAD caused by repeated anesthesia exposure. The *ire-1* branch of the UPR and ERAD pathways could be the target of melatonin to reduce the anesthesia-induced ER stress. Further studies are required to unravel how melatonin acts in mammal models and how the cascade may be related to AIN.

## Methods

*Caenorhabditis elegans* strains, wild-type N2 (WB Cat# WBStrain00000001, RRID: WBStrain00000001) and *zcls4[hsp-4::GFP]V* (WB Cat# WBStrain00034065, RRID: WBStrain00034065), and an *Escherichia coli* strain (OP50) were purchased from the Caenorhabditis Genetics Center (Minneapolis, MN, USA) and maintained at 20 °C as per the regular protocol described in our previous report^16^. All experiments were performed at 20 °C, unless indicated otherwise. Molecular biology chemicals were obtained from MERCK KOREA (Seoul, South Korea).

### Melatonin preparation, anesthesia exposure, and chemotaxis assay

Synchronized worms were divided into four groups, namely control, isoflurane, control-melatonin, and isoflurane-melatonin groups. The isoflurane and isoflurane-melatonin groups were exposed to isoflurane four times, at the first (L1), second (L2), third (L3), and fourth (L4) larval stages. The worms were anesthetized using 99.9% effective dose of isoflurane, which had been determined by our previous experiment. The duration of each exposure was 1 h, and the interval between each anesthesia was 3 to 4 h.

The worms of control-melatonin and isoflurane-melatonin groups were maintained in the melatonin-added nutrient growth medium (NGM) with OP50. Melatonin was diluted in the NGM to have the final concentrations of 3 nM, 10 nM, 30 nM, 100 nM, 300 nM, and 1000 nM. As a melatonin receptor inhibitor and a proteasome inhibitor, 300 μM luzindole or 50 μM MG132 was respectively added to the medium during the anesthetic exposure^47,49^.

In order to evaluate the behavioral effect due to repeated anesthesia exposure, a chemotaxis assay was performed as described previously^16^. Briefly, when synchronized worms became young adults, they were washed in S-basal medium and the worm pellet was located at the center of the chemotaxis plate. The number of worms found in each attractant or control site was counted, and the chemotaxis index was calculated according to the following formula: (number of worms at attractant site − number of worms at control site)/total number of worms × 100. The chemotaxis assay was performed with each melatonin concentration (Fig. S1) and 100 nM melatonin was chosen and used in subsequent experiments.

### Fluorescence imaging

Adult-stage worms, of the *zcls4[hsp-4::GFP]V* strain, were immobilized using 0.5 M sodium azide and mounted on an agar pad. Green fluorescence protein (GFP) expression was visualized using a ZEISS LSM 710 confocal microscope system (Oberkochen, Germany). Based on GFP expression in the control group, that of other three groups was quantified.

### RNA isolation and real-time polymerase chain reaction (PCR)

Total RNA was isolated from the adult worms of each group and RNA quality was assessed by measuring the absorbance ratio at 260–280 nm and at 260–230 nm. cDNA was synthesized using Maxima H minus first strand cDNA synthesis kit (THERMO FISHER SCIENTIFIC, Waltham, MA, USA). Real-time PCR was then performed using each primer, cDNA, and POWER SYBR GREEN PCR MASTER MIX (BIOSYSTEMS, Waltham, MA, USA). Primer sequences are listed in Table 1. Gene expression levels were normalized to pan-actin, and the ratio of expression was compared across the four groups.

**Table 1:** Forward and reverse primer sequences for real-time PCR.

*hsp-4*	Forward	CGTGGCAAACGCGTACTGTGATGAAGGAGC
	Reverse	CAGTTCATCATGATCCTCCGATTGCTCCTC
*ire-1*	Forward	ACAATGGCTAGTCAGCGAGG
	Reverse	CTTCTGGAGCAATCCAGCCA
*pek-1*	Forward	TGACATTGACACCGACGAGG
	Reverse	TGCCCGATGACCTTCTTGAC
*atf-6*	Forward	ATCGTTGCTCCTGCCTAGTG
	Reverse	TCAATTGGCCAGTCCCTGTC
*sel-1*	Forward	GTGGACGAGGGCTCAATCAA
	Reverse	AATGCATCGGCACTTCCTGA
*sel-11*	Forward	GCGTCTTCCACACCAACAAC
	Reverse	CCTAGAAGACGTGCTAGGCG
*cdc-48.1*	Forward	TGCTCACAATGTGGTTCGGA
	Reverse	GAACAACACGCAAGGAGCAG
*cdc-48.2*	Forward	GAGAAGCGTATCGTCTCGCA
	Reverse	TTAGTAGCGGCGATCACGAC
*pan-actin*	Forward	TCGGTATGGGACAGAAGGAC
	Reverse	CATCCCAGTTGGTGACGATA

### Western blot analysis

Worms were lysed in cold RIPA lysis buffer (BRA0500, BIOMAX; 25 mM Tris–HCl, pH 7.6, 150 mM NaCl, 1% NP-40, 1% sodium deoxycholate, and 1% sodium dodecyl sulfate) containing 100 mM iodoacetamide, and the worm lysates were quantified using a PIERCE BCA PROTEIN ASSAY KIT (THERMO FISHER SCIENTIFIC). Equal volumes of protein lysates were resolved by SDS-PAGE and transferred to polyvinylidene difluoride membranes. The membrane was blocked by 5% skim milk in a Tris-buffered saline with 0.1% Tween 20 (20 mM Tris, 500 mM sodium chloride, and 0.1% Tween 20, pH 7.5), and incubated for 1 h at 20 °C with primary anti-ubiquitin antibody (1:1000; ABCAM Cat# ab7254, RRID:AB_305802) and anti-actin antibody (1:5000; ABCAM Cat# ab14128, RRID:AB_300931), followed by horseradish peroxidase-conjugated secondary antibody (SANTA CRUZ BIOTECHNOLOGY Cat# sc-516102, RRID:AB_2687626). Signal was detected with a chemiluminescence kit (DG-W250, DoGen, Korea).

### Statistics

Data are expressed as the mean ± standard deviation, unless specified otherwise. Student’s t-test or one-way analysis of variance with Bonferroni t-test for post-hoc analysis was performed, as appropriate. All batches contained three plates in each group, and each plate included approximately 50–100 worms. The same assay was performed three times, unless otherwise stated. The SPSS software (ver. 21; IBM Co., Armonk, NY, USA) was used for statistical analyses and a P value < 0.05 was considered significant.

## Supplementary Information

Supplementary Figures.

## Data Availability

The datasets generated during and/or analyzed during the current study are only available from the corresponding author on reasonable request.
