# Supplementary material for: Melatonin reduces the endoplasmic reticulum stress and polyubiquitinated protein accumulation induced by repeated anesthesia exposure in Caenorhabditis elegans
Source: Sci Rep. 2022 Apr 6;12:5783. doi: 10.1038/s41598-022-09853-y (PMC8986834; doi:10.1038/s41598-022-09853-y)
Supplement: Supplementary file 1 — Supplementary Figures. [file 41598_2022_9853_MOESM1_ESM.docx]

**
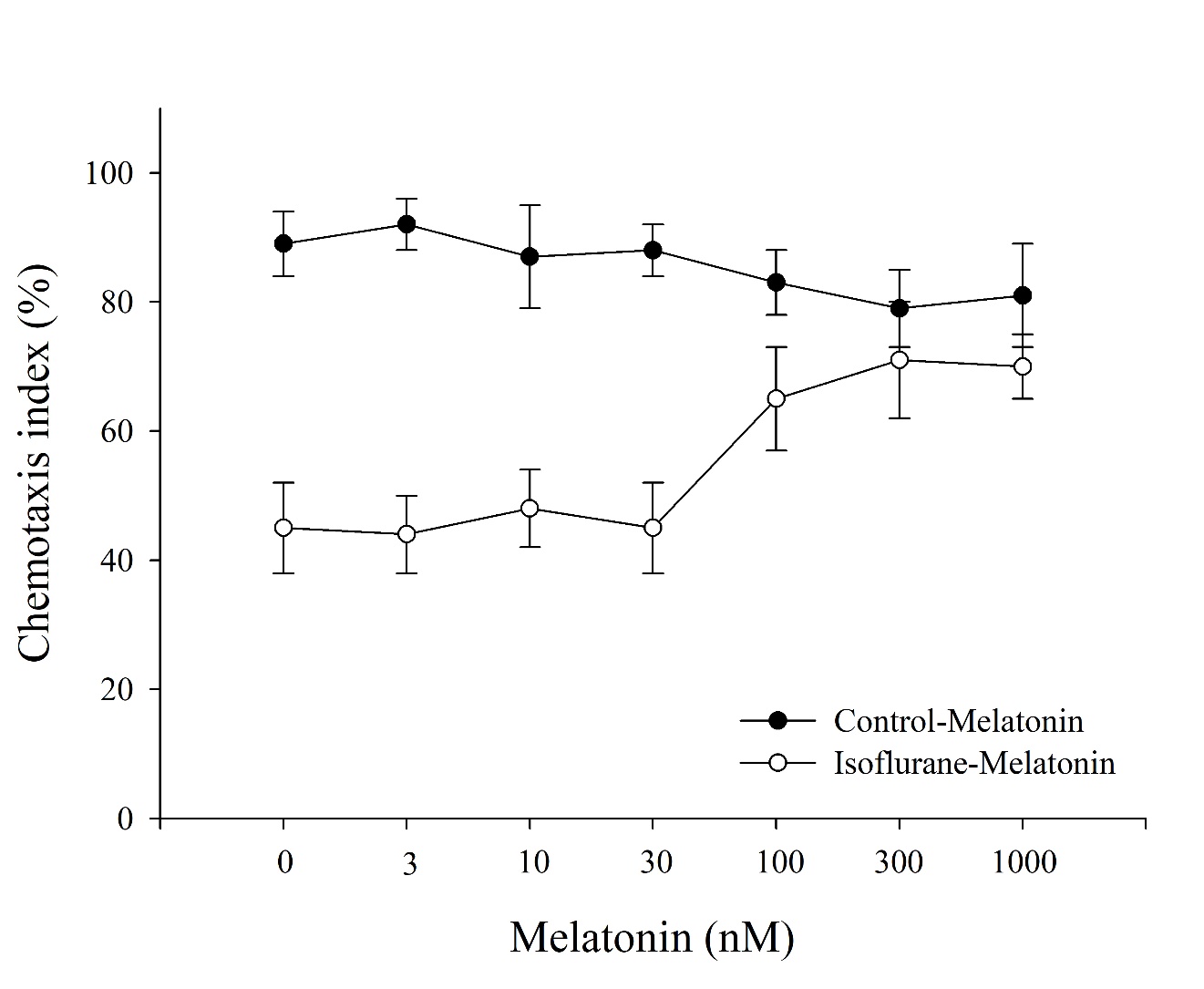
**

**Figure S1. Chemotaxis indices according to the melatonin concentration in *C. elegans***

The change of chemotaxis index in both groups: control-melatonin and isoflurane-melatonin group. The half maximal effective concentration (EC50) of melatonin was 93.1 nM. Thus, *C. elegans* was treated with 100 nM melatonin in the following experiments. All batches included two plates in each group and the same assay was performed five times.


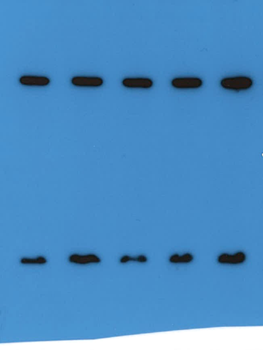


**Figure S2. The original version of western blot for GFP**


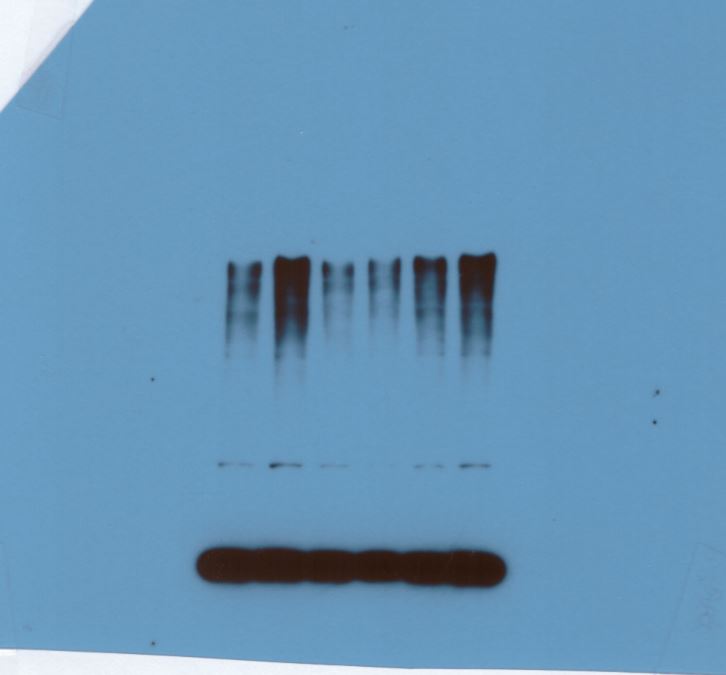
**Figure S3. The original version of western blot for ubiquitinated proteins**
